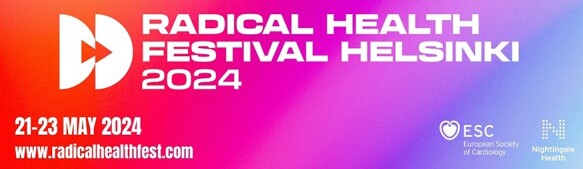# Radical Health Festival Helsinki 2024 preview: navigating the future of healthcare

**DOI:** 10.1093/ehjdh/ztae030

**Published:** 2024-04-23

**Authors:** Nurgül Keser, Joost Lumens, Lukasz Koltowski, Gerd Hindricks, Nico Bruining

**Affiliations:** Cardiology, Health Sciences University, Sultan Abdulhamid Han Training and Research Hospital, Istanbul, Turkey; CARIM School for Cardiovascular Diseases, Maastricht University Medical Center, Maastricht, The Netherlands; 1st Chair and Department of Cardiology, Medical University of Warsaw, Warsaw, Poland; German Heart Center of the Charite, Berlin, Germany; Department of Cardiology, Heart Vessel Institute, Thoraxcenter, Erasmus MC, Dr Molewaterplein 40, Rotterdam 3015 GD, The Netherlands

The Radical Health Festival Helsinki (RHFH) is a symbol of pan-European collaboration, aiming to revolutionize healthcare, improve outcomes, and ensure health system sustainability. In its inaugural year in 2023, RHFH drew over 1000 participants from 30+ countries. With engaging sessions and 160 speakers, the festival gained global media attention and was praised for its collaborative approach and dedication to healthcare transformation.

Building on the successful partnership between the European Society of Cardiology (ESC) and RHFH, we are pleased to announce the renewal of our collaboration. This ensures a seamless continuation of our partnership at the upcoming event, scheduled for 22–23 May 2024, in Helsinki. Professor Gerhard Hindricks, Chair of the ESC’s Digital Health Committee, highlights the creation of synergies and pathways in the digital health arena.

The ESC, a leading provider of cardiovascular education, recommendations, and resources for the global cardiology community, recognizes the strategic importance of digital health. Through dedicated tracks at ESC congresses, webinars, and platforms like the *European Heart—Journal Digital Health*, the ESC is at the forefront of digital health discourse. Collaborating with partners like RHFH positions the ESC in high-level discussions, fostering a diverse network crucial for navigating the evolving healthcare landscape.

The upcoming festival aims to surpass the success of the previous year, focusing on precision medicine and preventive strategies in healthcare. With the theme ‘Deploying Precision and Prevention at Scale,’ this year’s event requires vision, leadership, change management, and radical innovation.

Exploring this theme through three perspectives—innovation, people, and data—the festival aims to accelerate adoption, support entrepreneurship, update reimbursement processes, promote digital literacy, and integrate health and social care data for precise approaches to population health. Healthcare systems worldwide are currently facing unprecedented strain, with limited resources and overextension as major challenges. Aging populations, the increasing prevalence of cardiometabolic diseases, workforce shortages, rising healthcare costs, unsustainable financing systems, and healthcare inequities underscore the urgent need for transformative change. The debate on whether our current healthcare system adequately promotes preventive measures is gaining momentum. Radical Health Festival Helsinki serves as a platform for posing difficult questions to global leaders and fostering dialogue on reshaping healthcare. While artificial intelligence (AI)-driven pathways are improving clinical approaches, ensuring the safe implementation of AI initiatives remains a significant challenge. Digital health is transforming the roles of healthcare professionals, redefining patient–physician relationships, and emphasizing shared decision-making.

In today’s healthcare landscape, effective clinical intervention relies on personalized patient interaction and shared decision-making. Rapid innovation, particularly in AI, holds great promise for personalized health guidance. However, this also poses its own challenges. Navigating these innovations requires addressing systemic challenges while striking a delicate balance between disruption and adaptability.

Moreover, there is a global call for individuals to take greater responsibility for their health. True transformation occurs when individuals take control of their health, with clinicians serving as trusted partners. This shift emphasizes the need to prepare the next generation of healthcare professionals to succeed in this evolving era.

As the lead clinical partner of the festival, the ESC is well positioned to address these urgent issues. Their sessions will cover a wide range of topics, including digital health innovations, AI in cardiology, connected health, digital twin technology, and patient empowerment. This strategic initiative aligns with the ESC’s broader plan to establish European standards and provide training in digital health technologies, ensuring cardiovascular health professionals are equipped for the future.

Radical Health Festival Helsinki 2024 is the result of collaborative efforts from more than 20 key European contributing partners. This extensive network is prepared to advance the ESC’s digital health mission. Join us for an exploration of ideas and networking prospects focused on the theme ‘Deploying Prevention and Precision at Scale’. Visit the website to learn more about RHFH 2024.